# A study on the processing technology for Rhizoma Coptidis

**DOI:** 10.1186/s12896-021-00731-5

**Published:** 2022-01-14

**Authors:** Yunhong Wang, Weihan Qin, Yujie Yang, Hui Bai, Jirui Wang, Xiaomei Zhang, Yanlei Guo, Lei Hua, Yong Yang

**Affiliations:** 1 Department of Institute of Pharmacochemistry of Traditional Chinese Medicine, Chongqing Academy of Chinese Materia Medica, No. 34 of Nanshan Street, Nanan District, Chongqing, 400065 China; 2 Chongqong University of Education, Chongqing, 400067 China

**Keywords:** Wine coptis, Processing technology, Box–Behnken, Alkaloids, Yunlian

## Abstract

**Background:**

The present study intends to optimize the processing technology for the wine-processing of Rhizoma Coptidis, using alkaloids as indicators.

**Method:**

In the present study, the Box–Behnken design method was adopted to optimize the processing technology for Rhizoma Coptidis, using the alkaloid component quantities as the index. 100 g of Rhizoma Coptidis slices and 12.5 g of Rhizoma Coptidis wine were used. After full mixing, box-Behnken design method was used to optimize the processing time, processing temperature and processing time of coptis chinensis by taking alkaloid content as index. After mixing well, these components were fried in a container at 125 °C for 6 min and exhibited good parallelism.

**Results:**

The content of alkaloids in coptis chinensis was the highest after roasting at 125 °C for 6 min. The characteristic components were berberine hydrochloride, and the relative content was about 15.96%. And showed good parallelism. The effective components of Rhizoma Coptidis were primarily alkaloids.

**Conclusion:**

The optimized processing technology for Rhizoma Coptidis is good.

## Background

Rhizoma Coptidis is the dry rhizome of *Coptis chinensis*, *Coptis deltoidea*, or *Coptis teeta* of the Ranunculaceae family [1–3], known as "Weilian", "Yalian" and "Yunlian," respectively. Rhizoma Coptidis has a bitter and cold nature and has the effect of clearing away heat, drying dampness, purging fire, and detoxifying [4, 5]. Purging fire and detoxifying is a term used in Traditional Chinese medicine (TCM). TCM believes that diseases can be divided into cold syndrome and heat syndrome, requiring symptomatic treatment. In Traditional Chinese medicine, diseases manifested by heat syndrome are usually called heat toxicity, so there is a treatment of reducing heat to relieve heat toxicity in Traditional Chinese medicine. It's called catharsis and detoxification. This is the nature of traditional Chinese medicine (TCM) under the guidance of the theory of TCM. These Chinese medical terms cannot be well translated into English, and therefore, pinyin is used. According to the theory of TCM, bitter cold herbs usually have a thin Qi and a heavy taste (coptis tastes bitter, gas cold, for the big bitter and big cold goods, cannot be overserved, long served, raw good at clearing the heart fire. After processing, wine coptis can mitigate the bitter cold in the raw food, lead up medicine, good head of the fire). Qi and heavy taste are also characteristics of Traditional Chinese medicine, and are classified under the guidance of traditional Chinese medicine theory. Taste has five tastes namely sour bitter sweet salty. When processed with wine, it is possible to both make use of the pungent hot properties and delivery function of the wine to alleviate the bitter cold nature of this herb, stop it from hurting the spleen and stomach, and allow it to be cold without stagnating. The combination of the hot and the cold allows it to play a better role in clearing heat and purging fire [6, 7].

Usually, Chinese medicinal materials are the original plants, animals and minerals, which can not be directly used for the treatment of diseases. They need to be processed before they can be used, which is called yinpian, and the processing method and process is called processing. The commonly used processing methods are steaming, boiling and stir-frying. Sometimes it is necessary to add different ingredients for processing, such as wine, wheat bran, ginger, etc. Processing has the effect of alleviating and changing the curative effect. In this study, *Coptis chinensis* belongs to cold medicine in Traditional Chinese medicine, and the wine used in processing is hot, which is processed to ease its bitter cold. In clinical practice, there are various methods for processing Rhizoma Coptidis with wine, e.g., adding rice wine (12.5 g of rice wine for every 100 g of Rhizoma Coptidis); mixing well; after the rice wine has been absorbed, placing it in a container preheated to 125 °C and frying it for 6 min; taking it out; and allowing it to cool. However, there is no consensus over the best processing method [8–12]. Therefore, the present study intends to optimize the processing technology for the wine-processing of Rhizoma Coptidis, using alkaloids as indicators. The aim is to suggest a method for obtaining great quantities of the components with a high degree of purity.

## Methods

### Chromatographic conditions

**Fig. 1:**
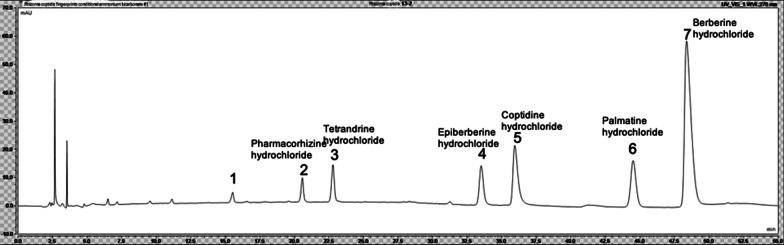
High-performance liquid chromatogram. The numbered peaks represent: 2. Pharmacorhizine hydrochloride, 3. Tetrandrine hydrochloride, 4. Epiberberine hydrochloride, 5. Coptidine hydrochloride, 6. Palmatine hydrochloride, and 7. Berberine hydrochloride

**Fig. 2:**
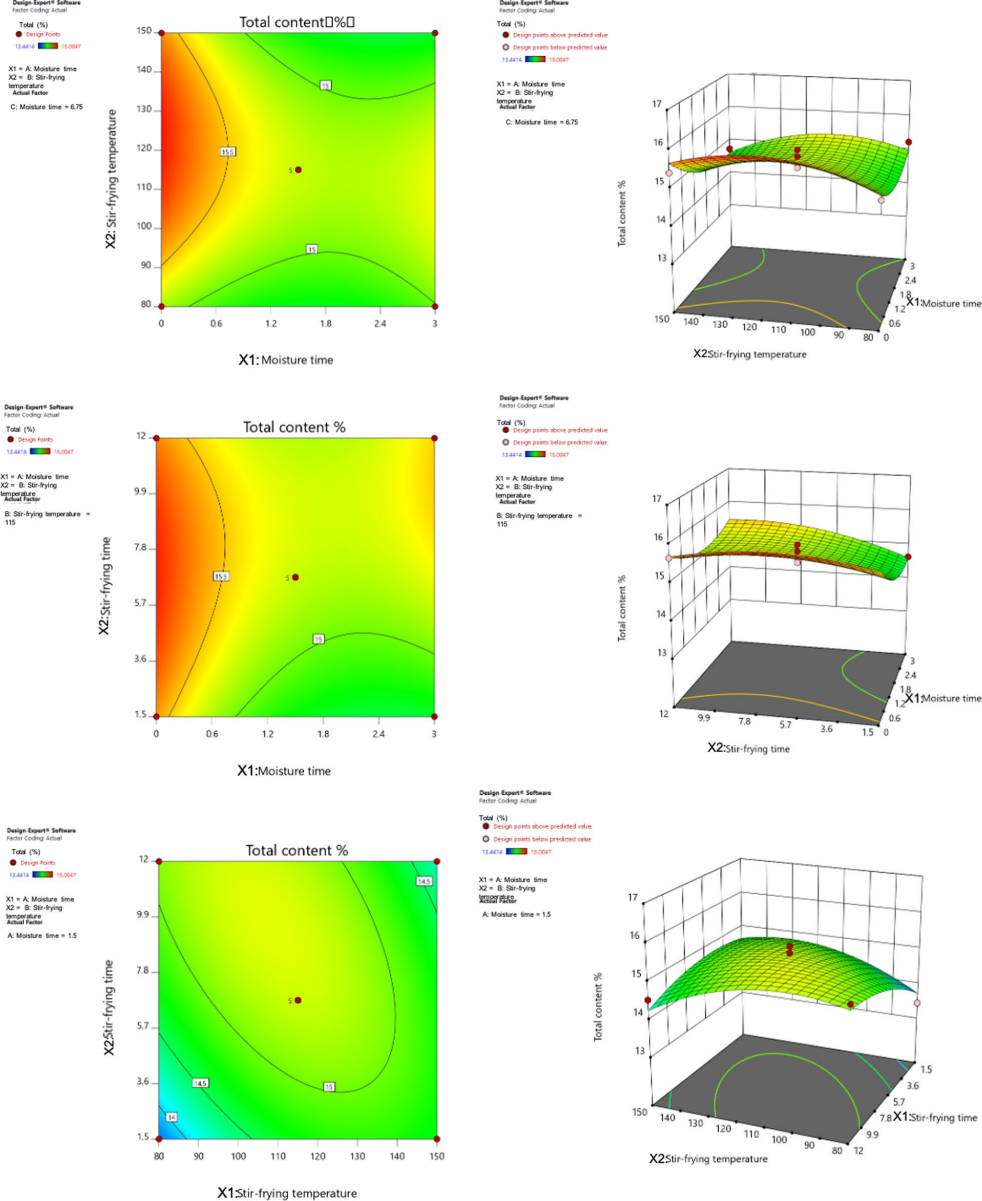
Contour and response surface

**Fig. 3:**
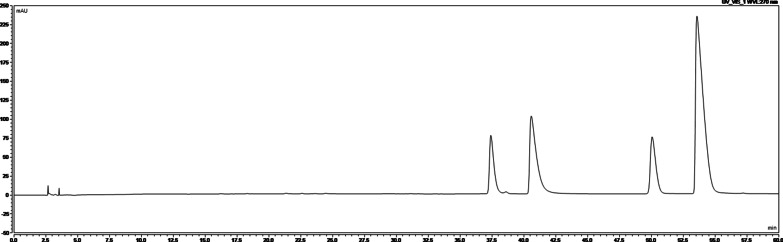
Chromatogram of reference substances: 1. Epaperberine, 2. Coptidine, 3. Palmatine, and 4. Berberine

The chromatographic conditions used in the present study were as follows: The column used was the Welch Xtimate® C18 (4.6 × 250.0 mm, 3 μm), the detection wavelength was 270 nm, and the column temperature was 30 °C. In addition, the loading volume was 10 μL, and the herb underwent gradient elution with acetonitrile-30 mmol/L ammonium bicarbonate (containing 0.7% ammonia and 0.1% triethylamine) as the mobile phase, with a flow rate of 1 mL/min. The specific chromatogram is shown in Figs. 1, and 3 shows the chromatogram of the reference substances (Figs. 2, 3).

### The preparation of the test solution

The Chinese medicine coptis chinensis is purchased from Chongqing Shizhu,Origin: Coptis chinensis Franch. Dry rhizomes. A powder passing a 10-mesh sieve of the herb to be tested was obtained, and 0.2 g was placed in a corked, conical flask, and precisely added with 50 ml of 50% acetonitrile-hydrochloric acid (100:1) mixed solution was added. The conical flask was tightly plugged, weighed, treated with ultrasound (power 250 W, frequency 40 kHz) for 30 min, left standing for cooling, and weighed. A 50% acetonitrile-hydrochloric acid (100:1) solution was added to make up the weight loss, and it was shaken and filtered using qualitative filter paper. Of the resultant filtrate, 2 mL was placed in a 10 mL measuring flask and a 50% acetonitrile-hydrochloric acid (100:1) solution was added until it reached the required weight. The test solution should be pretreated and filtered with a 0.22 µm membrane before injection into HPLC. It was shaken and filtered using a 0.22 μm microporous filter membrane. The resultant filtrate was the required solution.

## Results

### Single-factor investigation of the processing technology for the wine-processing of Rhizoma Coptidis

#### Investigation into the liquid absorption rate

Modern studies show that the following six alkaloids are mainly found in coptis chinensis, which are: Medicine stephania root alkali, hydrochloric acid Africa hydrochloric acid alkali, hydrochloric acid table berberine and hydrastine hydrochloric acid, hydrochloric acid, Martin, berberine hydrochloride (corresponding to peak in the chromatograph chart 2 ~ 7), but because of peak separation effect is good, and the area is larger, thus will peak respectively reference 1 ~ 7 as processing technology research, but because of these seven elements only berberine hydrochloride in standard material, Therefore, the chromatographic peak area of berberine hydrochloride was used as reference.

In the present study, 100 g of Rhizoma Coptidis was weighed, 500 ml of yellow wine (manufacturer: Chengdu Julong Biological Technology Co., LTD., raw materials and accessories: water, rice, caramel color; The alcohol is 10.0%vol; Total sugar: 15.0 g/L or less) was added, and the mixture was left to stand for one hour. The liquid was extracted, and the volume was measured (see Table 1). The results showed that the absorption rate of Rhizoma Coptidis was approximately 1 ml/g.

**Table 1:** Alcohol absorption capacity of Rhizoma coptidis

Weighing sample (g)	Add fluid volume (ml)	Residual fluid volume (ml)
100.00	500	400
100.00		405
100.00		405

#### Investigation into the liquid absorption time

According to the 2015 edition of the Chinese Pharmacopoeia, the procedures for the wine-processing of Rhizoma Coptidis are as follows: 100 g of Rhizoma Coptidis and 12.5 g of yellow wine (approximately 12.5 ml), and the amount of yellow wine should be far less than that of Rhizoma Coptidis. Therefore, in the present study, 100 g of Rhizoma Coptidis and 12.5 g of yellow wine were weighed and used. They were mixed well, and the absorption time was measured. The results revealed that the yellow wine can be completely absorbed in 3 min.

#### Investigation into the processing time

In the present study, 100 g of Rhizoma Coptidis and 12.5 g of yellow wine were weighed, mixed well, and stirred and fried at 80 °C and 150 °C. The elapsed time was recorded. The results showed that the herb had a well-processed appearance at 12 and 1.5 min, respectively.

#### Investigation into the type of yellow wine and the alcohol content

In the present study, 12.5 g of different types of yellow wine with varying alcohol contents were used. Each type of wine was mixed well with 100 g of Rhizoma Coptidis and fried at 115 °C for 7 min. The alkaloid content in each sample was then measured (see Table 2). The results revealed that the alkaloid content in the wine-processing of Rhizoma Coptidis processed using different types of wine with varying alcohol concentrations was similar.

**Table 2:** Alkaloid contents in different types of yellow rice wine and prepared alcoholic products (% by dry weight)

Variety and alcohol of yellow rice wine	Epiberberine (%)	Coptisine (%)	Palmatine (%)	Berberine (%)
Dry type,16%	1.33	2.26	2.23	7.95
Semi-dry,15%	1.29	2.22	2.21	7.98
Semi-Swee,10%	1.36	2.28	2.22	7.89
Sweet,11.5%	1.34	2.29	2.23	8.01
RSD%	2.22	1.37	0.44	0.65

### The processing technology for the wine-processing of Rhizoma Coptidis designed by Box–Behnken

#### Factor-level design

With the processing temperature, processing time, and moisture time as independent variables, seven characteristic peaks were measured to calculate the content percentages, using berberine hydrochloride as a reference. The sum of these seven characteristic peaks (Y) was used as the response value for the design. The experimental design and results are shown in Tables 3 and 4.

**Table 3:** Ranges of design factors of Box-behnken

Level	Moisture time/min *A*	Processing temperature/°C *B*	Processing time/min *C*
Low	0	80	1.5
High	3	150	12

**Table 4:** Box-behnken design test and results

No	*A*_1_/ min	*B*/°C	*C*/min	*Y1/*%	*Y2/*%	*Y3/*%	*Y4/*%	*Y5/*%	*Y6/*%	*Y7/*%	*Y*
1	3	150	6.75	0.18	0.39	0.58	1.41	2.28	2.13	7.77	
2	0	80	6.75	0.16	0.40	0.61	1.38	2.33	2.17	7.99	
3	1.5	80	1.5	0.15	0.39	0.52	1.26	2.14	1.95	7.03	
4	3	115	12	0.16	0.40	0.64	1.41	2.29	2.25	7.93	
5	1.5	80	12	0.17	0.43	0.65	1.35	2.36	2.23	8.01	
6	1.5	115	6.75	0.16	0.41	0.65	1.35	2.26	2.21	7.94	
7	1.5	115	6.75	0.20	0.42	0.66	1.49	2.31	2.22	7.83	
8	0	150	6.75	0.18	0.42	0.64	1.46	2.35	2.29	8.07	
9	3	115	1.5	0.17	0.41	0.64	1.38	2.19	2.23	7.61	
10	1.5	115	6.75	0.18	0.41	0.67	1.46	2.29	2.27	8.04	
11	1.5	150	12	0.17	0.40	0.64	1.33	2.14	2.20	7.65	
12	1.5	150	1.5	0.18	0.42	0.62	1.39	2.21	2.19	7.52	
13	3	80	6.75	0.19	0.42	0.64	1.43	2.36	2.21	7.98	
14	1.5	115	6.75	0.18	0.41	0.67	1.46	2.35	2.28	8.15	
15	0	115	12	0.18	0.42	0.68	1.47	2.35	2.38	8.16	
16	1.5	115	6.75	0.18	0.42	0.69	1.43	2.32	2.24	7.93	
17	0	115	1.5	0.19	0.42	0.67	1.45	2.51	2.33	8.42	
Predictive value				0.178	0.420	0.683	1.500	2.440	2.356	8.376	15.964

Y1% to Y7% are equally important

#### Model fit and variance analysis

The content of all processed products showed different degrees of decrease when compared with the original medicinal materials. However, since the active ingredients were alkaloids, the total content (%) was chosen as the evaluation index, with the aim of optimizing its maximum value. The obtained regression model equation is as follows: Y = 15.23 − 0.3026A + 0.0333B + 0.2334C − 0.2121AB + 0.1991AC − 0.4357BC + 0.3969A^2^ − 0.5237B^2^ − 0.2837C^2^, r^2^ = 0.8450. These results reveal that the goodness of fit of the model is good, and it can be used for the analysis and prediction of the processing technology. The processing temperature and processing time of B and C factors had no significant effect on the content of alkaloids. The analysis of variance revealed that within the test range, the influencing factors for the processing technology were as follows (in decreasing order of the magnitude of influence): moisture time, processing time, and processing temperature (see Table 5).

**Table 5:** Variance analysis

Source	Sum of Squares	df	F-value	*P*-value	
Model	4.36	9	0.4898	0.0350*	Significant
A-moisture time	0.7323	1	6.41	0.0392	
B- processing temperature	0.0089	1	0.0775	0.7888	
C- processing time	0.4358	1	3.81	0.0918	
AB	0.1800	1	1.57	0.2498	
AC	0.1585	1	1.39	0.2774	
BC	0.7592	1	6.64	0.0366	
A^2^	0.6634	1	5.80	0.0468	
B^2^	1.15	1	10.10	0.0155	
C^2^	0.3388	1	2.96	0.1288	
Residual	0.8001	7			
Lack of Fit	0.6581	3	6.18	0.0555	Not significant
Pure error	0.1420	4			
Cor total	5.16	16			

* indicates the significant value

#### Optimization and prediction of the response surface

The Design-Expert 11 software was used to draw the response surface and a two-dimensional contour map of the moisture time (min), processing temperature (°C), and processing time (min) of the wine-processing of Rhizoma Coptidis.

With Y-max as the goal, the processing technology was optimized, and the results were as follows: A = 0, B = 125.792, and C = 5.828. Considering the actual situation of the instruments and equipment, the best process was adjusted to the following: A = 0, B = 125, and C = 6. This means that 100 g of Rhizoma Coptidis and 12.5 g of yellow wine should be weighed, mixed well, and stirred and fried at 125 °C for 6 min.

Using the optimal conditions, the value of Y1–Y7% and the value of Y are predicted as: Y1/% = 0.178, Y2% = 0.420,Y3% = 0.683,Y4% = 1.500,Y5 = 2.440,Y6 = 2.356,Y7 = 8.376,Y = 15.964.

#### Verification test

In the present study, according to the proposed process, three batches of the processed product were prepared in parallel. The results revealed that the values of Y (%) for the three batches of the processed products were 15.41, 15.38, and 15.31 (see Table 6). The data in Table 6 reveal that the optimized process in the present study exhibits good parallelism.

**Table 6:** Verify test results

	*Y*% (predicted value)	*Y*% (measured value)
1	15.964	15.41
2		15.38
3		15.31

## Discussion

The TCM theory considers the following: the wine-processing of Rhizoma Coptidis can reduce its bitter-cold nature and can lead the medicine upward, and modern studies have revealed that the active ingredients are alkaloids [13–15]. In addition, both processed and unprocessed Rhizoma Coptidis have the same material basis and similar alkaloid content but different effects. This needs to be investigated using pharmacodynamics [16–20]. In the present study, the Box–Behnken design method was adopted, and the alkaloid content was used as the index to optimize the processing technology for the wine-processing of Rhizoma Coptidis. The results revealed that the main active ingredients in the wine-processing of Rhizoma Coptidis are alkaloids.

Limitations: The following limitations exist in the present study. First, the pharmacological effect of the Rhizoma Coptidis after wine roasting is not clear in the present study. Since the effective components of Rhizoma Coptidis are alkaloids, in the present study, the processing technology for Rhizoma Coptidis was optimized using alkaloids as indicators. However, according to the theory of TCM, wine-processing can reduce the bitter cold nature of Rhizoma Coptidis and change its pharmacological effect. This team plans to conduct further studies on the pharmacological effects of the wine-processing of Rhizoma Coptidis. In addition, the clinical efficacy of Rhizoma Coptidis after processing still needs to be further verified through clinical research.

Compared with traditional processing, the processing process was datalized and the processing end point was more objective. The traditional culture of processing traditional Chinese medicine is mostly inherited by the master and apprentice, and the end point of processing is subjective judgment of human beings, which cannot form a unified standard. In this study, digital process parameters were used to specify the processing endpoint, which can combine traditional process with modern technology to a certain extent.

## Conclusion

The main effective components of the wine-processing of Rhizoma Coptidis are alkaloids. The processing technology for the wine-processing of Rhizoma Coptidis is good and worthy of clinical popularization.

## Data Availability

The datasets used and/or analysed during the current study available from the corresponding author on reasonable request.

## Abbreviation

TCM: Traditional Chinese medicine
